# A dataset for the analysis of antibody response to glycan alpha-Gal in individuals with immune-mediated disorders

**DOI:** 10.12688/f1000research.27495.1

**Published:** 2020-11-24

**Authors:** José de la Fuente, José Miguel Urra, Marinela Contreras, Iván Pacheco, Elisa Ferreras-Colino, Ernesto Doncel-Pérez, Isabel G. Fernández de Mera, Margarita Villar, Carmen M. Cabrera, Cesar Gómez Hernando, Eduardo Vargas Baquero, Javier Blanco García, Javier Rodríguez Gómez, Alberto Velayos Galán, Francisco Feo Brito, Elisa Gómez Torrijos, Alejandro Cabezas-Cruz, Christian Gortázar

**Affiliations:** 1 SaBio, Instituto de Investigación en Recursos Cinegéticos IREC, Ciudad Real, 13005, Spain; 2 Department of Veterinary Pathobiology, Center for Veterinary Health Sciences, Oklahoma State University, Stillwater, OK, 74078, USA; 3 Immunology Department, Hospital General Universitario de Ciudad Real, Ciudad Real, 13005, Spain; 4 School of Medicine, Universidad de Castilla la Mancha (UCLM), Ciudad Real, 13005, Spain; 5 Interdisciplinary Laboratory of Clinical Analysis, Interlab-UMU, Regional Campus of International Excellence Campus Mare Nostrum, University of Murcia, Murcia, 30100, Spain; 6 Hospital Nacional de Parapléjicos, Servicio de Salud de Castilla La Mancha, Toledo, 45071, Spain; 7 Biochemistry Section, Faculty of Science and Chemical Technologies, and Regional Centre for Biomedical Research (CRIB), University of Castilla-La Mancha, Ciudad Real, 13071, Spain; 8 Hospital Virgen de la Salud, Toeldo, 45001, Spain; 9 Servicio de Neurología, Hospital General La Mancha Centro, Alcázar de San Juan, 13600, Spain; 10 Allergy Section, General University Hospital of Ciudad Real, Ciudad Real, 13005, Spain; 11 UMR BIPAR, INRAE, ANSES, Ecole Nationale Vétérinaire d'Alfort, Université Paris-Est, Maisons-Alfort, 94700, France

**Keywords:** alpha Gal, immune response, antibody, allergy, tick, coronavirus, COVID-19, Guillain-Barré syndrome, alpha-Gal syndrome

## Abstract

Humans evolved by losing the capacity to synthesize the glycan Galα1-3Galβ1-(3)4GlcNAc-R (α-Gal), which resulted in the development of a protective response mediated by anti-α-Gal IgM/IgG/IgA antibodies against pathogens containing this modification on membrane proteins. As an evolutionary trade-off, humans can develop the alpha-Gal syndrome (AGS), a recently diagnosed disease mediated by anti-α-Gal IgE antibodies and associated with allergic reactions to mammalian meat consumption and tick bites. However, the anti-α-Gal antibody response may be associated with other immune-mediated disorders such as those occurring in patients with COVID-19 and Guillain-Barré syndrome (GBS). Here, we provide a dataset (209 entries) on the IgE/IgM/IgG/IgA anti-α-Gal antibody response in healthy individuals and patients diagnosed with AGS, tick-borne allergies, GBS and COVID-19. The data allows correlative analyses of the anti-α-Gal antibody response with factors such as patient and clinical characteristics, record of tick bites, blood group, age and sex. These analyses could provide insights into the role of anti-α-Gal antibody response in disease symptomatology and possible protective mechanisms.

## Introduction

The gene coding for α-1,3-galactosyltransferase ( *α1,3GT*) was inactivated in old-world monkeys, an evolutionary adaptation that resulted in the production of high antibody titers against glycan Galα1-3Galβ1-(3)4GlcNAc-R (α-Gal) ( Galili, 2015). Previous results showed that up to 1–5% of the circulating IgM/IgG found in healthy individuals are directed against α-Gal ( Macher & Galili, 2008). Bacteria in the human gut microbiome express *α1,3GT* genes to produce α-Gal epitopes ( Montassier *et al*., 2020), suggesting that natural anti-α-Gal antibodies are produced in response to gut microbiota ( Bello-Gil *et al*., 2019; Galili *et al*., 1988; Mañez *et al*., 2001; Yilmaz *et al*., 2014). This evolutionary adaptation has been associated with the protective response of anti-α-Gal IgM/IgG antibodies against pathogens containing this modification on membrane proteins ( Galili, 2018; Hodžić *et al*., 2020). In contrast, the presence of α-Gal in tick salivary glycoproteins and glycolipids ( Araujo *et al*., 2016; Cabezas-Cruz *et al*., 2018; Chinuki *et al*., 2016; Crispell *et al*., 2019) and tick cement ( Villar *et al*., 2020) induces anti-α-Gal IgE antibodies that mediate delayed anaphylaxis to mammalian meat consumption and immediate anaphylaxis to tick bites, xenotransplantation and certain drugs such as cetuximab ( Cabezas-Cruz *et al*., 2019; Commins *et al*., 2009; Contreras *et al*., 2020; de la Fuente *et al*., 2019a; de la Fuente *et al*., 2020; Fischer *et al*., 2016; Levin *et al*., 2019; Mateos-Hernández *et al*., 2017; Platts-Mills *et al*., 2020; Steinke *et al*., 2015; van Nunen *et al*., 2007).

Factors that may affect the antibody response to α-Gal include but are not limited to age, repeat consumption of certain food and meats of different origin or innards with higher α-Gal content, exposure to tick bites, ABO blood group, co-occurring disorders and exposure to cats and other pets ( Cabezas-Cruz *et al*., 2017; Cabezas-Cruz *et al*., 2019; Commins, 2016; Commins *et al*., 2014; de la Fuente *et al*., 2020a; Fischer *et al*., 2014; Fischer *et al*., 2016; Morisset *et al*., 2012; Platts-Mills *et al*., 2020; Wölbing *et al*., 2013). Additionally, the anti-α-Gal-specific IgE response has been associated with other diseases such as atopy, coronary artery disease and atherosclerosis ( Gonzalez-Quintela *et al*., 2014; Wilson *et al*., 2017; Wilson *et al*., 2019). Furthermore, α-Gal-mediated innate and adaptive immune response mechanisms have been associated with protection against pathogen infection in various animal models ( Hodžić *et al*., 2020). However, little is known about the influence of anti-α-Gal immune response on immune-mediated disorders such as those occurring in patients with COVID-19 and Guillain-Barré syndrome (GBS).

These results raise questions and hypothesis regarding the role of α-Gal-mediated immune responses in disease symptomatology and possible protective mechanisms ( de la Fuente *et al*., 2019b; de la Fuente *et al*., 2020b; Pacheco *et al*., 2020; Urra *et al*., 2020). Consequently, to advance in addressing these questions and hypothesis, here we provide data on the IgE/IgM/IgG/IgA anti-α-Gal antibody response in healthy individuals and patients diagnosed with AGS, tick-borne allergies, GBS and COVID-19. These data contribute to correlative analyses of the anti-α-Gal antibody response with factors such as patient and clinical characteristics, record of tick bites, blood group, age and sex. These analyses could provide insights into the role of anti-α-Gal antibody response in disease symptomatology and protection against immune-mediated disorders.

## Materials and methods

Essential methods used for the generation of the dataset ( de la Fuente *et al*., 2020) were described in Urra *et al*. (2020) with additional information in Pacheco *et al*. (2020) and Doncel-Pérez *et al*. (2020).

### Patients and healthy individuals

A retrospective case-control study was conducted in patients suffering from COVID-19 admitted to the University General Hospital of Ciudad Real (HGUCR), Spain from March 1 to April 15, 2020. The infection by SARS-CoV-2 was confirmed in all patients included in the study by the real-time reverse transcriptase-polymerase chain reaction (RT-PCR) assay from Abbott Laboratories (Abbott RealTime SARS-COV-2 assay, Abbott Park, Illinois, USA) from upper respiratory tract samples after hospital admission. Clinical features, as well as laboratory determinations were obtained from patient's medical records. The patients were grouped as hospital discharge, hospitalized and intensive care unit ( Urra *et al*., 2020). Patients were hospitalized for developing a moderate-severe clinical condition with radiologically demonstrated pneumonia and failure in blood oxygen saturation. Patients with acute respiratory failure who needed mechanical ventilation support were admitted to a hospital ICU. The patients were discharged from the hospital due to the clinical and radiological improvement of pneumonia caused by the SARS-CoV-2, along with the normalization of analytical parameters indicative of inflammation, such as C-reactive protein (CRP), D-Dimer and blood cell count ( Urra *et al*., 2020). Samples from asymptomatic COVID-19 cases with positive anti-SARS-CoV-2 IgG antibody titers but negative by RT-PCR were collected in May 22–29, 2020 and included in the dataset ( Urra *et al*., 2020). Samples from healthy individuals (individuals without record of tick bites and allergic reactions) and patients diagnosed with tick-borne allergic reactions (AGS, anaphylaxis or urticaria) were collected prior to COVID-19 pandemic in April 2019 ( Pacheco *et al*., 2020). The use of human peripheral blood serum samples from healthy individuals and patients diagnosed with tick-borne allergic reactions was done with their written informed consent in compliance with the Helsinki Declaration. Nursing personnel at the General University Hospital of Ciudad Real, Spain, extracted blood samples. Samples and data from patients with GBS included in this dataset were provided by the BioB-HVS, integrated into the Spanish National Biobanks Network. All samples were processed following standard operating procedures with the appropriate approval of the Ethical and Scientific Committees (Toledo Hospitable Complex 29012014-No17, University Hospital of Ciudad Real C-352 and SESCAM C-73).

### Preparation of serum samples

For the preparation of serum samples, a sterile tube without anticoagulant was used to collect blood samples. The blood from each patient and the healthy individual was maintained in standing position at room temperature (RT) for clotting (20–30 min) and centrifuged at 1,500 × g for 20 min at RT. Serum was collected and conserved at -20°C until used for analysis.

### Determination of antibody titers against α-Gal

For ELISA, high absorption capacity polystyrene microtiter plates were coated with 50 ng of BSA coated with α-Gal (BSA-α-Gal, thereafter named α-Gal; Dextra, Shinfield, UK) per well in carbonate-bicarbonate buffer (Sigma-Aldrich, St. Louis, MO, USA). After an overnight incubation at 4°C, coated plates were washed one time with 100 µl/well PBS with 0.05% Tween 20 (PBST) (Sigma-Aldrich), blocked with 100 µl/well of 1% human serum albumin (HAS) in PBST (Sigma-Aldrich) for 1 h at RT and then washed four times with 100 µl/well of PBST. Human serum samples were diluted 1:100 in PBST with 1% HAS and 100 µl/well were added into the wells of the antigen-coated plates and incubated for 1 h at 37°C. Plates were washed four times with PBST and 100 µl/well of goat anti-human immunoglobulins-peroxidase IgG (FC specific) (Cat. No. I2136), IgM (µ-chain specific) (Cat. No. I1636), and IgE (ɛ-chain specific) (Cat. No. I6284) secondary antibodies (Sigma-Aldrich) diluted 1:1000, v/v in blocking solution were added and incubated for 1 h at RT. Plates were washed four times with 100 µl/well of PBST and 100 µl/well of 3,3,´5,5-tetramethylbenzidine TMB (Promega, Madison, WI, USA) were added and incubated for 20 min at RT. Finally, the reaction was stopped with 50 µl/well of 2 N H _2_SO _4_ and the O.D. was measured in a spectrophotometer at 450 nm. The average of two technical replicates per sample was used for analysis after background (coated wells incubated with PBS and secondary antibodies) subtraction. 

### Statistical analysis

**Figure 1.**
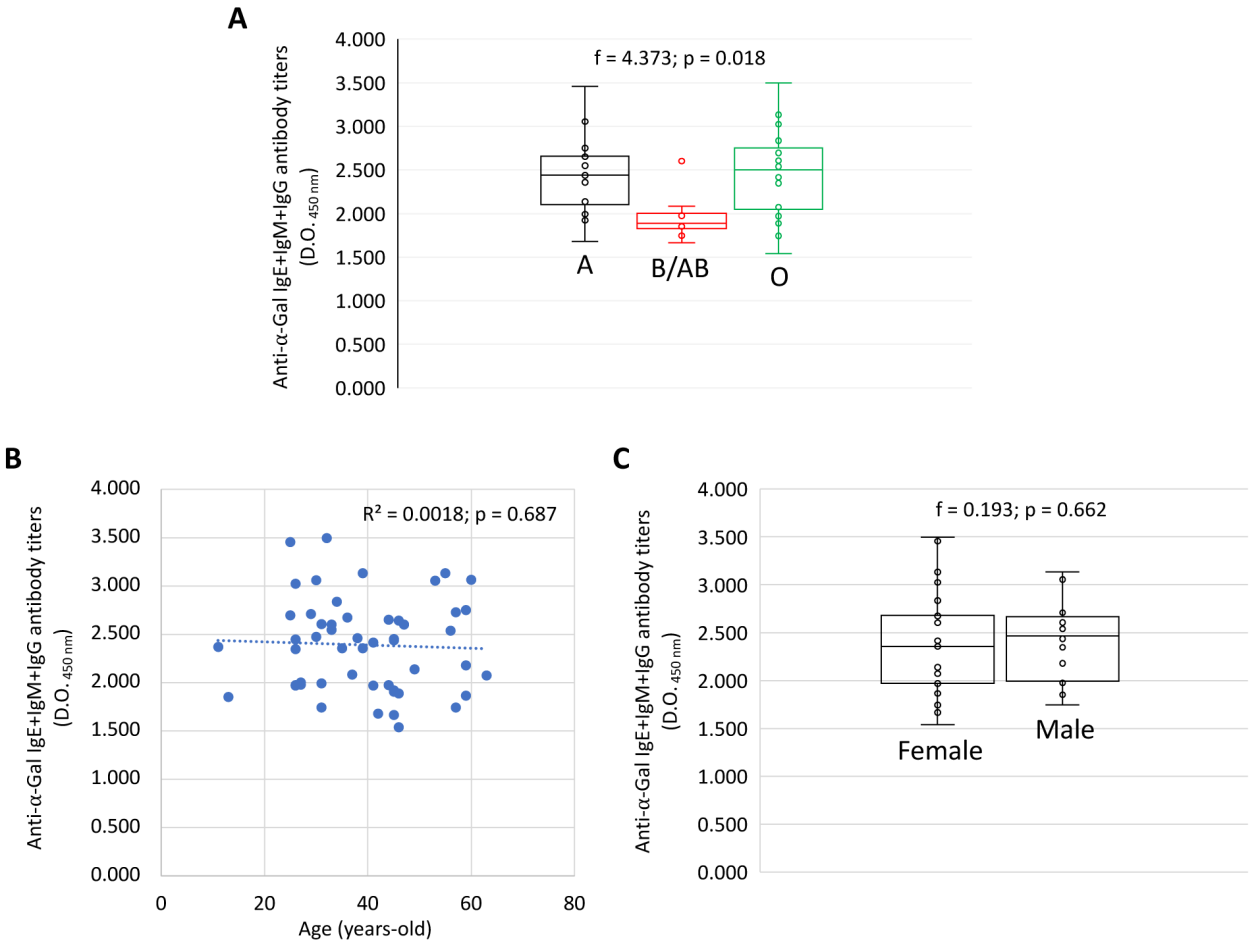
An example of the effect of certain factors such as ( **A**) blood group, ( **B**) age and ( **C**) sex on the antibody response to α-Gal in healthy individuals. Anti-α-Gal IgE, IgM and IgG antibody titers were determined by ELISA. ( **A, C**) The ELISA O.D. at 450 nm values were compared for each Ig by one-way ANOVA test (p < 0.05). ( **B**) A Spearman Rho correlation analysis (p < 0.01) was conducted between anti-α-Gal IgE, IgM and IgG antibody titers and age. Correlation coefficient (R ^2^) is shown.

Anti-α-Gal IgE, IgM and IgG antibody titers (O.D. at 450 nm values) were compared for each Ig by one-way ANOVA test (p < 0.05) ( https://www.socscistatistics.com/tests/anova/default2.aspx) ( Figure 1A and 1C). A Spearman Rho correlation analysis (p < 0.01; https://www.socscistatistics.com/tests/spearman/default2.aspx) was conducted between anti-α-Gal IgE, IgM and IgG antibody titers and age ( Figure 1B).

### Dataset validation

The dataset ( de la Fuente *et al*., 2020) was validated in studies reported by Urra *et al*. (2020), Pacheco *et al*. (2020) and Doncel-Pérez *et al*. (2020). Additionally, a comparative analysis was conducted between the IgE+IgM+IgG antibody response to α-Gal and blood groups ( Figure 1A), age ( Figure 1B) and sex ( Figure 1C) in healthy individuals (n = 75) to illustrate lower antibody titers in blood group B/AB individuals as previously reported ( Cabezas-Cruz *et al*., 2017) but no differences regarding age and sex, which have been reported before as factors affecting the antibody response to α-Gal, infection and vaccination ( Buonomano *et al*., 1999; Giefing-Kröll *et al*., 2015; Wang *et al*., 1995).

The main limitation of the dataset is sample size for some factors (i.e. age, sex or blood group), which were not disclosed by all individuals, and anti-α-Gal IgA antibody titers that could be considered in the analysis ( Mateos-Hernández *et al*., 2020; Urra *et al*., 2020). 

## Data availability

### Underlying data

Harvard Dataverse: A dataset for the analysis of antibody response to glycan alpha-Gal in individuals with immune-mediated disorders. https://doi.org/10.7910/DVN/RBU2VR ( de la Fuente *et al*., 2020).

This dataset contains characteristics and serum antibody levels of the individuals included in the study and was used in analyses reported in publications by Urra *et al*. (2020), Pacheco *et al*. (2020) and Doncel-Pérez *et al*. (2020).

Data are available under the terms of the Creative Commons Zero "No rights reserved" data waiver (CC0 1.0 Public domain dedication).
