# Supplementary material for: A dataset for the analysis of antibody response to glycan alpha-Gal in individuals with immune-mediated disorders
Source: F1000Res. 2021 May 25;9:1366. Originally published 2020 Nov 24. [Version 2] doi: 10.12688/f1000research.27495.2 (PMC8361808; doi:10.12688/f1000research.27495.2)
Supplement: Peer review history

# Peer review: Reviewer response for version 2

**DOI:** 10.5256/f1000research.57019.r91810

**Published:** 2021-08-12

**Authors:** Mona Mahmoud

**Affiliations:** 1 Parasitology and Animal Diseases Department, National Research Center, Giza, Egypt

The article is scientifically sound. The data includes a dataset of anti-alphaGal IgM, IgG, and IgE levels in the serum of healthy people and patients, including COVID-19 patients, tick bite patients, and patients with Guillain-Barré syndrome. The rationale and methods are stated clearly, and the dataset is provided in a way that is both accessible and convenient.

Are sufficient details of methods and materials provided to allow replication by others?

Yes

Is the rationale for creating the dataset(s) clearly described?

Yes

Are the datasets clearly presented in a useable and accessible format?

Yes

Are the protocols appropriate and is the work technically sound?

Yes

Reviewer Expertise:

immuoparasitology, Molecular biology

I confirm that I have read this submission and believe that I have an appropriate level of expertise to confirm that it is of an acceptable scientific standard.

# Peer review: Reviewer response for version 2

**DOI:** 10.5256/f1000research.57019.r86098

**Published:** 2021-07-20

**Authors:** Tilo Biedermann

**Affiliations:** 1 Department of Dermatology and Allergology, Technical University of Munich, Munich, Germany; 2 Clinical Unit Allergology, Hemholtz Zentrum Munchen, German Research Centre for Environmental Health, Neuherberg, Germany

**Response to 1**

 The presentation of the data set in Figure 1 is still not clear to me. What is the rational to sum up OD _450_ values of α-Gal IgM, IgG and IgE ELISAs? Since Figure 1 is an example to demonstrate the possible usage of the data set, it should in my opinion consist of analyzes with a scientific value. Thus, I would recommend to exemplary show one of the isotypes instead of the summation of all measured isotypes.

**Response to 2**

 I agree that different factors such as tick bites correlate with higher α-Gal IgE titers. However, a rational explanation for the high α-Gal IgE titers in a large proportion of healthy controls is still missing (atopy? History of repetitive tick bites?). The article by Pacheco *et al.* mentioned by the authors (DOI: 10.1016/j.ttbdis.2021.101651) shows a quantitative analysis of α-Gal IgE (ImmunoCAP) in addition to the ELISA assay results. However, while, as expected and also in accordance with previous publications by others (DOI: 10.1111/all.13400), only a small proportion of healthy, asymptomatic individuals (15%) were positive for α-Gal IgE in the ImmunoCAP analysis, almost all sera analyzed by ELISA showed α-Gal IgE levels above the cut-off for positivity (OD _450_ = 0.3) defined by the authors in another publication (DOI: 10.1038/emm.2016.164), regardless of the tick bite or allergy history of the respective individual. Additionally, the correlation analysis in Supplementary Figure 1 (Pacheco et al.) is not clear to me. What is the x axis corresponding to (number of individuals)? Is it not the case that each individual corresponding to one of the different allergy-type reaction groups is defined by one individual symbol in the graph? If I understood correctly, the formula to convert antibody titers into kU/l is based on a correlation of OD _450_ values generated by ELISA and kU/l values of the corresponding samples determined by ImmunoCAP. Could you please share the corresponding correlation with me in order to reproduce the results shown in Supplementary Figure 1C? Using the formula to determine α-Gal IgE titers as stated in Pacheco et al., only OD _450_ values higher than 2.7 result in kU/l values higher than the cut-off of 0.35 kU/l (which is not fitting to the cut-off of OD _450_ of 0.3 defined for positivity of the ELISA). Although I agree that the relation between α-Gal IgE levels of healthy individuals and AGS patients fits the literature (higher OD _450_ in AGS and anaphylactic patients compared to healthy), I am still missing an explanation for the surprisingly high OD _450_ values of α-Gal IgE in a large proportion of healthy individuals, especially if the cut-off for positivity of the α-Gal IgE ELISA is set to OD _450 _0.3.

Are sufficient details of methods and materials provided to allow replication by others?

Yes

Is the rationale for creating the dataset(s) clearly described?

Yes

Are the datasets clearly presented in a useable and accessible format?

Partly

Are the protocols appropriate and is the work technically sound?

No

Reviewer Expertise:

NA

I confirm that I have read this submission and believe that I have an appropriate level of expertise to state that I do not consider it to be of an acceptable scientific standard, for reasons outlined above.

## References

[1] : Characterization of the anti-α-Gal antibody profile in association with Guillain-Barré syndrome, implications for tick-related allergic reactions.*Ticks Tick Borne Dis*.12(3) : 10.1016/j.ttbdis.2021.10165110165110.1016/j.ttbdis.2021.10165133465663

[2] : Immunoprofile of α-Gal- and B-antigen-specific responses differentiates red meat-allergic patients from healthy individuals.*Allergy*.2018;73(7) : 10.1111/all.134001525-153110.1111/all.1340029319188

[3] : Effect of blood type on anti-α-Gal immunity and the incidence of infectious diseases. *Experimental & Molecular Medicine*.2017;49(3) : 10.1038/emm.2016.16410.1038/emm.2016.164PMC538255728280265

# Response

**Published:** 2021-07-21

**Authors:** Jose de la Fuente

**Affiliations:** Instituto de Investigación en Recursos Cinegéticos IREC, Spain

We appreciate reviewer comments, but consider that the information addressing these comments was provided in the previous response and revised manuscript. Exposure to ticks is always a variable that is difficult to fully address although this question was included in data collection as reflected in the database associated to the paper. Therefore, we have no further revisions in response to these comments.

# Peer review: Reviewer response for version 2

**DOI:** 10.5256/f1000research.57019.r86099

**Published:** 2021-05-26

**Authors:** Jacques Le Pendu

**Affiliations:** 1 CRCINA, Université de Nantes, Inserm, Nantes, France

No further comments to make.

Are sufficient details of methods and materials provided to allow replication by others?

Yes

Is the rationale for creating the dataset(s) clearly described?

Yes

Are the datasets clearly presented in a useable and accessible format?

Yes

Are the protocols appropriate and is the work technically sound?

Yes

Reviewer Expertise:

Glycobiology, Host-pathogens interactions, histo-blood group antigens

I confirm that I have read this submission and believe that I have an appropriate level of expertise to confirm that it is of an acceptable scientific standard.

# Peer review: Reviewer response for version 1

**DOI:** 10.5256/f1000research.30388.r84326

**Published:** 2021-05-18

**Authors:** Tilo Biedermann

**Affiliations:** 1 Department of Dermatology and Allergology, Technical University of Munich, Munich, Germany; 2 Clinical Unit Allergology, Hemholtz Zentrum Munchen, German Research Centre for Environmental Health, Neuherberg, Germany

The data note presented by José de la Fuente and colleagues contains α-Gal specific antibody titers of IgE, IgM, IgG isotypes in the serum and, for some individuals, IgA isotype in saliva of healthy individuals as well as patients with tick bite-associated symptoms, Guillain-Barré syndrome and COVID-19 infection, respectively. The dataset was already used in a published study by the authors correlating α-Gal specific antibodies and COVID-19 disease symptoms (Urra *et al*. 2020, ^1^). Although the dataset is given in an easily accessible format (Excel sheet) and methods are adequately described, I have some major concerns in regard to the benefit of the data for other research applications which are listed below.

1. The authors validate their dataset using comparative analysis of antibody titers of healthy individuals with blood group, age and sex. What is the rational to use a summation of α-Gal IgG, IgM and IgE antibody levels for these analyses? Since different antibody isotypes are associated with certain immunological functions (effector functions mediated by Fc part of the antibody) and certain diseases are associated with certain antibody isotypes (e.g. AGS and α-Gal IgE), a summation of different antibody isotype levels is in my opinion not correct and does not provide meaningful scientific insights. Additionally, since the entries for the key characteristics are incomplete for a large proportion of the individuals included, the used comparative analysis is not generally applicable for the presented dataset and thus not appropriate for its validation.
2. The OD _450_ values and thus the titers of α-Gal specific IgE are unexpectedly high in almost half of the healthy individuals. Is there an explanation for α-Gal IgE titers in serum of healthy individuals which are as high as in AGS patients or individuals with tick-bite associated allergic reactions? Can these individuals for certain be classified as “healthy” or how can sensitization to α-Gal be explained (atopy)? In my opinion, the ELISA data presented in this manuscript requires validation due to the unexpected and controversial high titers of α-Gal specific IgE antibodies in “healthy” individuals e.g. by using α-Gal ImmunoCAP or the commercially available α-Gal IgE ELISA Kit.
3. As also stated by the authors as main limitation of their dataset, key characteristics such as age, sex and blood group are missing for a large proportion of the entries. This incompleteness significantly limits the benefit of the dataset for other research questions.

Are sufficient details of methods and materials provided to allow replication by others?

Yes

Is the rationale for creating the dataset(s) clearly described?

Yes

Are the datasets clearly presented in a useable and accessible format?

Partly

Are the protocols appropriate and is the work technically sound?

No

Reviewer Expertise:

Immunology, Allergy, Dermatology.

I confirm that I have read this submission and believe that I have an appropriate level of expertise to state that I do not consider it to be of an acceptable scientific standard, for reasons outlined above.

## References

[1] : The antibody response to the glycan α-Gal correlates with COVID-19 disease symptoms.*J Med Virol*.93(4) : 10.1002/jmv.265752065-207510.1002/jmv.2657533009829PMC7675678

# Response

**Published:** 2021-05-18

**Authors:** Jose de la Fuente

**Affiliations:** Instituto de Investigación en Recursos Cinegéticos IREC, Spain

Thanks for your comments to our paper. In response to your comments:

 The data note presented by José de la Fuente and colleagues contains α-Gal specific antibody titers of IgE, IgM, IgG isotypes in the serum and, for some individuals, IgA isotype in saliva of healthy individuals as well as patients with tick bite-associated symptoms, Guillain-Barré syndrome and COVID-19 infection, respectively. The dataset was already used in a published study by the authors correlating α-Gal specific antibodies and COVID-19 disease symptoms (Urra *et al*. 2020, ^1^). Although the dataset is given in an easily accessible format (Excel sheet) and methods are adequately described, I have some major concerns in regard to the benefit of the data for other research applications which are listed below.

 1. The authors validate their dataset using comparative analysis of antibody titers of healthy individuals with blood group, age and sex. What is the rational to use a summation of α-Gal IgG, IgM and IgE antibody levels for these analyses? Since different antibody isotypes are associated with certain immunological functions (effector functions mediated by Fc part of the antibody) and certain diseases are associated with certain antibody isotypes (e.g. AGS and α-Gal IgE), a summation of different antibody isotype levels is in my opinion not correct and does not provide meaningful scientific insights. Additionally, since the entries for the key characteristics are incomplete for a large proportion of the individuals included, the used comparative analysis is not generally applicable for the presented dataset and thus not appropriate for its validation.

**Response**: We agree with reviewer on the differences between antibody isotypes as disclosed in the Introduction of the paper. However, Figure 1 is only an example to illustrate the effect of some factors such as blood group, age and sex on the total antibody response to α-Gal. This is why total antibody titers for subtypes IgE, IgM and IgG were added to illustrate these correlations or the absence of it. In no case we are using these data for validation or immunologically related analyses. For this purpose, please refer to these papers published using this dataset and with analyses using different antibody isotypes:

 Urra JM, Ferreras-Colino E, Contreras M, Cabrera CM, Fernández de Mera IG, Villar M, Cabezas-Cruz A, Gortázar C, de la Fuente J. The antibody response to the glycan α-Gal correlates with COVID-19 disease symptoms. J Med Virol. 2021 Apr;93(4):2065-2075. doi: 10.1002/jmv.26575.

 Hodžić A, de la Fuente J, Cabezas-Cruz A. COVID-19 in the Developing World: Is the Immune Response to α-Gal an Overlooked Factor Mitigating the Severity of Infection? ACS Infect Dis. 2020 Dec 11;6(12):3104-3108. doi: 10.1021/acsinfecdis.0c00747.

 Doncel-Pérez, E., Contreras, M., Gómez Hernando, C., Vargas Baquero, E., Blanco García, J., Rodríguez Gómez, J., Velayos Galán, A., Cabezas-Cruz, A., Gortázar, C., de la Fuente, J. 2020. What is the impact of the antibody response to glycan alpha-Gal in Guillain-Barré syndrome associated with SARS-CoV-2 infection? Merit Research Journal of Medicine and Medical Sciences (MRJMMS) 8: 730-737. https://digital.csic.es/bitstream/10261/230769/1/whatinfect.pdf

 Pacheco I, Fernández de Mera IG, Feo Brito F, Gómez Torrijos E, Villar M, Contreras M, Lima-Barbero JF, Doncel-Pérez E, Cabezas-Cruz A, Gortázar C, de la Fuente J. Characterization of the anti-α-Gal antibody profile in association with Guillain-Barré syndrome, implications for tick-related allergic reactions. Ticks Tick Borne Dis. 2021 May;12(3):101651. doi: 10.1016/j.ttbdis.2021.101651.

 2. The OD _450_ values and thus the titers of α-Gal specific IgE are unexpectedly high in almost half of the healthy individuals. Is there an explanation for α-Gal IgE titers in serum of healthy individuals which are as high as in AGS patients or individuals with tick-bite associated allergic reactions? Can these individuals for certain be classified as “healthy” or how can sensitization to α-Gal be explained (atopy)? In my opinion, the ELISA data presented in this manuscript requires validation due to the unexpected and controversial high titers of α-Gal specific IgE antibodies in “healthy” individuals e.g. by using α-Gal ImmunoCAP or the commercially available α-Gal IgE ELISA Kit.

**Response**: This is an interesting observation that has been reported in other studies. However, factors such as tick bites or allergy do correlate with higher IgE antibody titers (see figure 2 in Pacheco et al. Ticks Tick Borne Dis. 2021 May;12(3):101651. doi: 10.1016/j.ttbdis.2021.101651). For the validation of the ELISA with ImmunoCAP please see Supplementary Figure A1 in this paper by Pacheco et al. with correlation analysis between different allergy-type reactions to tick bites and anti-α-Gal IgE antibody response using a Spearman Rho (rs) correlation analysis (p < 0.01) conducted between allergy-type reactions and anti-α-Gal IgE antibody titers determined by ELISA (O.D. at 450 nm) and converted to kU/l. Positive anti-α-Gal IgE levels were considered at cut-off value of 0.35 kU/l. Due to scope of Data note papers, we cannot disclose all this information in the paper, but can be referred to in the revised paper.

 3. As also stated by the authors as main limitation of their dataset, key characteristics such as age, sex and blood group are missing for a large proportion of the entries. This incompleteness significantly limits the benefit of the dataset for other research questions.

 Response: We acknowledge these limitations of the dataset that are due to ethic issues in some of the studies. Nevertheless, we consider that this information is useful for the scientific and medical community interested in the study of the antibody response to α-Gal.

# Peer review: Reviewer response for version 1

**DOI:** 10.5256/f1000research.30388.r75352

**Published:** 2020-12-04

**Authors:** Jacques Le Pendu

**Affiliations:** 1 CRCINA, Université de Nantes, Inserm, Nantes, France

The data note presents a dataset of levels of anti-alphaGal IgM, IgG and IgE in the serum of healthy individuals and of patients, including COVID-19 patients, patients with tick bites and Guillain Barré syndrome patients. The rationale and methods are clearly presented and the dataset is given in an easily accessible and convenient format.

 I would only like to see a clarification concerning the exact alphaGal-BSA antigen that was used for coating. The structure of the oligosaccharide should be given.

Are sufficient details of methods and materials provided to allow replication by others?

Yes

Is the rationale for creating the dataset(s) clearly described?

Yes

Are the datasets clearly presented in a useable and accessible format?

Yes

Are the protocols appropriate and is the work technically sound?

Yes

Reviewer Expertise:

Glycobiology, Host-pathogens interactions, histo-blood group antigens

I confirm that I have read this submission and believe that I have an appropriate level of expertise to confirm that it is of an acceptable scientific standard.

# Response

**Published:** 2020-12-04

**Authors:** Jose de la Fuente

**Affiliations:** Instituto de Investigación en Recursos Cinegéticos IREC, Spain

Thanks for your positive feedback to our paper. In response to your question, the alphaGal-BSA antigen used for coating the ELISA plates was Galα1-3Gal-BSA (3 atom spacer) (Product Code: NGP0203; https://www.dextrauk.com/products/neoglycoproteins/gala1-3gal-series-neoglycoproteins/product/288-gala1-3gal-bsa-3-atom-spacer).
